# A Magnetic Field SPR Sensor Based on Temperature Self-Reference

**DOI:** 10.3390/s21186130

**Published:** 2021-09-13

**Authors:** Xinwei Mo, Jiangtao Lv, Qiang Liu, Xiaoxiao Jiang, Guangyuan Si

**Affiliations:** 1 College of Information Science and Engineering, Northeastern University, Shenyang 110004, China; mxw18333560736@163.com (X.M.); liuqiang@neuq.edu.cn (Q.L.); jxx316@mail.ustc.edu.cn (X.J.); 2 Institute of Control Engineering, Northeastern University at Qinhuangdao, Qinhuangdao 066004, China; 3 Melbourne Centre for Nanofabrication, Victorian Node of the Australian National Fabrication Facility, Clayton, VIC 3168, Australia; guangyuansi.si@nanomelbourne.com

**Keywords:** photonic crystal fiber, surface plasmon resonance, magnetic fluid

## Abstract

In this paper, a novel D-shaped photonic crystal fiber sensor for simultaneous measurements of magnetic field and temperature is proposed and characterized. Based on the surface plasmon resonance theory, the D-shaped flat surface coated with a gold layer is in direct contact with magnetic fluid to detect magnetic field, and one of the relatively small air holes near the fiber core is filled with polydimethylsiloxane (PDMS) to sense temperature. The realization of measuring the magnetic field and temperature separately through two channels depends on the fact that the magnetic field only changes the refractive index of the magnetic fluid, but has no effect on the refractive index of PDMS. The refractive index of the magnetic fluid and PDMS can be affected by temperature at the same time. The sensor designed in this work can separate the variations of the magnetic field and temperature simultaneously, therefore solving the cross-sensitivity problem to further improve the magnetic field sensitivity. When the thickness of the gold film is 50 nm and the radius of the filling hole is 0.52 *µm*, the magnetic field sensitivity and the temperature sensitivity of magnetic field sensor based on temperature self-reference can reach 0.14274 nm/Oe and −0.229 nm/°C, respectively.

## 1. Introduction

Surface plasmon resonance (SPR) is a kind of physical optical phenomenon occurring at metal–dielectric interfaces [1,2,3]. When an interface of negative and positive dielectric constant materials is irradiated with light, there are both evanescent waves and plasmon waves on the surface of the metallic material. When the plasmon waves and evanescent waves meet the phase matching conditions (at a specific wavelength), the coupling resonance phenomenon occurs [4]. At this time, the energy of incident light is rapidly absorbed by electrons, and the reflectivity obviously decreases. Because the SPR phenomenon is extremely sensitive to small changes of the dielectric constant on the surrounding media of the metal film, high sensitivity sensing [5] is enabled, which can be widely used for biochemical applications.

Photonic crystal fibers (PCFs), also known as porous or micro-structured fibers, can guide a light beam to pass through the pores and achieve the purpose of axial light transmission. The cladding of PCF is generally composed of silicon dioxide (SiO_2_) and air holes parallel to the axis. PCF can selectively introduce different shapes and arrangements of holes in the cladding and core [6,7]. All the holes can be filled with various functional materials, including solid, liquid and gas [8,9,10]. The flexible structures and diversity of fillers make PCF have more excellent characteristics than traditional fibers. D-shaped PCF refers to a plane on the side of PCF, which can be used to detect environmental parameters based on the principle of evanescent wave absorption [11,12,13]. 

Magnetic field sensors have been extensively investigated in terms of their principles of operation and the performance characteristics under different applications [12]. In order to obtain high fidelity outputs, sensitivity and selectivity are two key characteristics for magnetic field sensors [13]. Optical fiber magnetic field sensors possess many advantages, such as small volume, anti-interference, high sensitivity and accuracy, corrosion resistance and so on, showing great potential in real-time and remote sensing of the magnetic field [14,15,16]. Furthermore, the combination of D-shaped photonic crystal fibers and SPR can be utilized to enhance its magnetic field sensing performance and sensitivity, and solve the problem of cross sensitivity of temperature and magnetic field by filling temperature sensitive materials [17,18,19]. Yang [20] and coworkers proposed a SPR sensor based on D-shaped micro-structured optical fiber (MOF) to realize the simultaneous measurements of refractive index (RI) and temperature. When the gold layer thickness is 40 nm, the sensitivities of temperature and RI reach the maximum value of 2 nm/°C and 2400 nm/RIU. Dong et al. [21] used a D-shaped fiber modal interferometer with magnetic fluid (MF) for magnetic field and temperature detection with a high sensitivity of 99.68 pm/Oe and −77.49 pm/°C, respectively. Nevertheless, the measuring range of the magnetic field of the sensor is only 0~21 Oe, and the cross-sensitivity complications will arise when the magnetic fluid is used to survey the temperature and magnetic field synchronously.

In this paper, we propose an SPR based D-shaped photonic crystal fiber sensor to measure magnetic field and temperature simultaneously. The optical fiber flat surface is coated with a gold film and covered with magnetic fluid to detect the magnetic field, and the temperature sensing can be performed by filling a gold-plated air hole in the cladding layer with a thermal sensitive material. The transmission spectrum of the proposed sensor has two resonance dips that have different sensitivities to external magnetic field and temperature, which means the sensor can realize simultaneous measurements of the above two parameters. In addition, the detection sensitivity of the sensor is significantly improved due to the padding of magnetic fluid and thermally sensitive materials.

## 2. Working Principles

**Figure 1:**
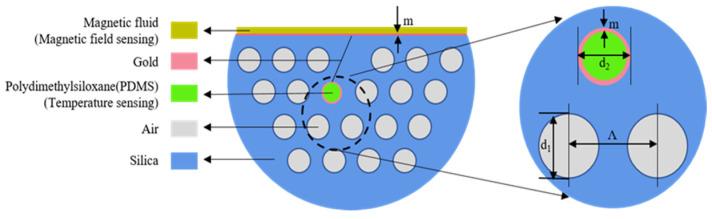
Cross section of the D-type photonic crystal fiber integrated with a gold film, magnetic fluid and PDMS.

The cross section of the SPR based D-shaped photonic crystal fiber magnetic field and temperature sensor is shown in Figure 1. The cladding of the fiber is composed of three layers of air holes, and the lattice constant is Λ = 2 *μm*. The diameter of air holes d_1_ is fixed at 1.4 *μm*, except the target pore with the diameter d_2_ = 1.04 *μm*. The optical fiber side is polished to form a flat plane, on which a gold film with a thickness of m = 50 nm is deposited, and the same thickness m of gold film has been plated on the surface of the target hole as well. Gold films are introduced as the SPR layers to excite surface plasmon polaritons (SPPs) and covered by PDMS and magnetic fluid at the target hole and flat, respectively. The air hole filled with PDMS is defined as Channel 1, and the magnetic fluid covering part is Channel 2. The optical transmission characteristics of the sensor are analyzed by finite element method and the perfectly matched layer (PML) is selected as the boundary condition in the simulation analysis process. The physical field is used to control the grid and the element size is conventional.

Since the complex permittivity of metals is related to the ambient temperature, the influence of temperature should be considered. Sellmeier dispersion model is used to illustrate the relationship between RI and wavelength. For the designed sensor, PCF is made of fused silica, and the thermo-optic coefficient and expansion coefficient of pure silica are set to 7.8 × 10^−6^/°C and 5.5 × 10^−7^/°C, respectively. The RI is obtained by using the Sellmeier equation and can be calculated by [22]:

(1) $$n^{2}\left(\lambda^{2}\right)=1+\frac{B_{1}\lambda^{2}}{\lambda^{2}-C_{1}}+\frac{B_{2}\lambda^{2}}{\lambda^{2}-C_{2}}+\frac{B_{3}\lambda^{2}}{\lambda^{2}-C_{3}}$$

where B_i_ (i = 1,2,3) and C_j_ (j = 1,2,3) are Sellmeier’s coefficients. The complex permittivity of gold films can be described by Drude model, which is expressed as [23]:

(2) $$\varepsilon_{Au}\left(T\right)=\varepsilon_{\infty }-\frac{\omega_{d}{\left(T\right)}^{2}}{\omega^{2}-\omega \omega_{c}\left(T\right)i}$$

where $\varepsilon_{Au}\left(T\right)$ is the complex permittivity of gold, $\varepsilon_{\infty }$ represents the complex permittivity at high frequencies, $\omega =2\pi_{c}/\lambda$ is the frequency of free space light, $\omega_{d}\left(T\right)$ and $\omega_{c}(T)$ denote the plasmon frequency and damping frequency, respectively. PDMS has great advantages of perfect mechanical properties, easy processing, low absorption loss, high thermo-optical coefficient and Poisson’s ratio. With increasing temperatures, the RI of PDMS will decrease, and the transmission spectrum will move towards the short wavelength range. In addition, the RI of PDMS and SPP mode will decrease. Both the changes will make the resonance point move to the short wavelengths. The relationship between the RI and temperature can be described as:

(3) $$n=n_{0}+\frac{dn}{dT}\left(T-T_{0}\right)$$

where *n*_0_ is the RI of PDMS at room temperature *T*_0_, $\frac{dn}{dT}$ represents the thermo-optic coefficient.

MF has been widely applied in optical fiber magnetic field sensors because of its adjustable magnetic index [24]. The RI of magnetic fluid varies with magnetic field and temperature. Langevin function [25] can be used to describe the change of RI of magnetic fluid under the influence of temperature and magnetic field strength:

(4) $$n_{MF}=\left[n_{s}-n_{0}\right]\left[\coth(\partial \frac{H-H_{c,n}}{T}\right)-\frac{T}{\partial \left(H-H_{c,n}\right)}]+n_{0}$$

where *n_s_* is the saturation value of the magnetic fluid RI, ∂ represents the fitting coefficient, *H_c,n_* and *n*_0_ denote the critical magnetic field strength and the RI of magnetic fluid when the external magnetic field *H* is less than *H_c,n_*. In this paper, the refractive index of MF is 1.3592, the thermo-optic and magneto-optic coefficients are −2.4 × 10^−4^/°C and 4.98 × 10^−5^/Oe, respectively. When the external temperature and magnetic field change, the RI of the filled sensitive material will also alter, which means the SPR loss peak shifts to different wavelengths. Therefore, the magnetic field and temperature can be measured by calculating the offset of the resonance wavelength. When the external magnetic field or temperature changes, the RI of the sample filled in the two channels will change, resulting in the variety of the resonance coupling phenomenon between the metal surface plasmon mode and the core mode. In other words, the SPR loss will change. The matrix relationship among resonant wavelength shift, temperature change and magnetic field change can be defined as follows:

(5) $$\left(\begin{array}{c} \Delta \lambda_{1}\\ \Delta \lambda_{2} \end{array}\right)=\left(\begin{array}{cc} \frac{\Delta \lambda_{1T}}{\Delta T} & \frac{\Delta \lambda_{1H}}{\Delta H}\\ \frac{\Delta \lambda_{2T}}{\Delta T} & \frac{\Delta \lambda_{2H}}{\Delta H} \end{array}\right)\left(\begin{array}{c} \Delta T\\ \Delta H \end{array}\right)=\left(\begin{array}{cc} K_{1T} & K_{1H}\\ K_{2T} & K_{2H} \end{array}\right)\left(\begin{array}{c} \Delta T\\ \Delta H \end{array}\right)$$

where *λ*_i,j_ (i = 1,2; j = *T*, *H*) is the variation of resonance wavelength, which can be used to detect the change of temperature ΔT and magnetic field intensity ΔH, *K*_1*T*_ and *K*_1*H*_ are the temperature sensitivity and magnetic field sensitivity of Channel 1, respectively. *K*_2*T*_ and *K*_2*H*_ are the same parameters of Channel 2.

Taking the temperature and magnetic field sensitivity of the above channels into the sensitivity matrix, the determinant of the matrix should not be equal to 0. Therefore, the sensitivity matrix can be inverted to obtain the sensing matrix for calculating the change of temperature and magnetic field intensity. Finally, according to the sensor matrix, the external environmental variables can then be detected:

(6) $$\left(\begin{array}{c} \Delta T\\ \Delta H \end{array}\right)={\left(\begin{array}{cc} K_{1T} & K_{1H}\\ K_{2T} & K_{2H} \end{array}\right)}^{-1}\left(\begin{array}{c} \Delta \lambda_{1}\\ \Delta \lambda_{2} \end{array}\right)$$

## 3. Results and Discussion

**Figure 2:**
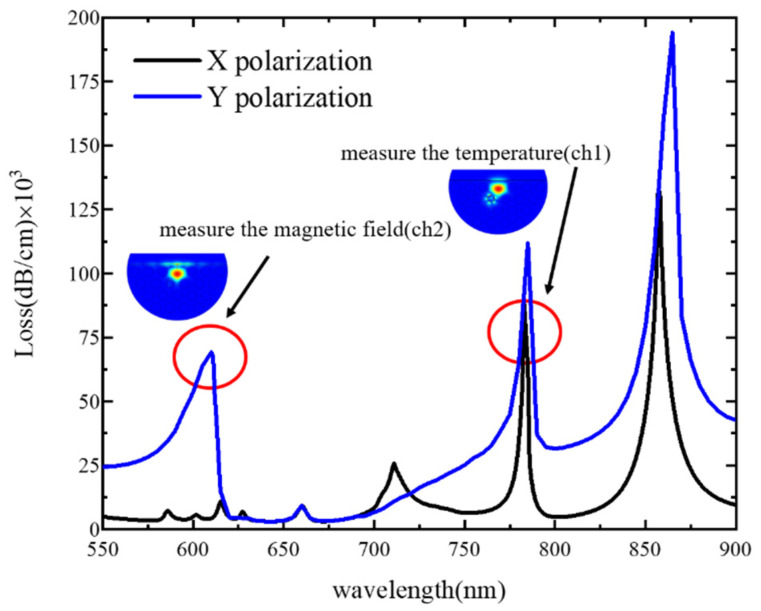
The loss spectrum of core mode X polarization and Y polarization.

When the temperature is 25 ℃, the magnetic field intensity change is 0 Oe, the diameter of the air hole in the cladding is d_1_ = 1.4 *μm*, the filling hole diameter is d_2_ = 1.04 *μm*, the lattice constant is Λ = 2 *μm*, and the thickness of the gold-plated film is m = 50 nm. We performed calculations by using COMSOL Multiphysics to verify the detection capability of the sensor, and then calculated the loss spectrum as shown in Figure 2.

As plotted in Figure 2, there are three peaks for X polarization. Because the PDMS in Channel 1 is not affected by the magnetic field intensity, the first and second peaks are only temperature sensitive and the loss of the second peak is higher. The SPPs in the second peak of X polarization are selected as the SPP modes of Channel 1. There are three peaks for Y polarization, and the second and third peaks are sensitive to temperature and magnetic field, while the first peak is only sensitive to magnetic field, so the SPPs at the first peak in Y polarization are selected as the SPP modes of Channel 2. In Channel 2, the SPP modes appear near the gold film on the polishing surface. The energy loss of the core mode reaches the maximum and the SPR effect is excited. Thus, part of the energy of the core mode is coupled to the SPP modes of Channel 2, and bright spots appear at the junction of the Channel 2 metal film and the analyte, which has a strong field distribution. In Channel 1, the SPP modes appear near the air hole of the gold film. The energy loss of the core mode reaches the maximum and the SPR effect is excited. Part of the energy of the core mode is coupled to the SPP modes of Channel 1, and bright spots appear at the junction of Channel 1 metal film and analyte, which also has a strong field distribution. Therefore, the measurements of two parameters can be realized by these two loss peaks. When the SPR effect is excited, the energy limiting ability of the core is weakened, and part of the energy of the core will be coupled to the metal SPP modes. Therefore, the loss peak of the core mode will appear at the resonance wavelength.

**Figure 3:**
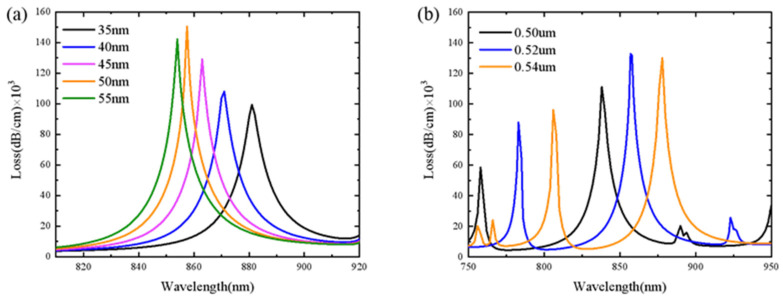
The loss spectrum with (**a**) different thicknesses of gold film and (**b**) different radii of the filled holes.

The thickness variation of the gold film is from 35 nm to 55 nm with 5 nm step size, and the results are plotted in Figure 3a. With the increase in gold film thickness, the loss spectrum shifts to left and the resonance wavelength exhibits a blueshift. We choose to set the thickness of the gold film to 50 nm for the higher peak loss and stronger SPP modes at the interface between the gold film and sensitive material. The contact area between the metal film and the dielectric material and the distance between the metal film and the sensor core will change with the radius of Channel 1, so the radius of Channel 1 affects the sensitivity of the sensor. The influence of hole radius on the loss spectrum is shown in Figure 3b. Under the condition that other parameters remain unchanged, we take three cases into account (the radius of the filling hole is 0.50 *μm*, 0.52 *μm* and 0.54 *μm* respectively). As illustrated in Figure 3b, the loss spectrum shifts to right and the resonance wavelength shows a redshift with the increase in the radius of Channel 1 hole. When the radius is 0.50 *μm*, 0.52 *μm* and 0.54 *μm*, the loss peaks, respectively correspond to 111,199.1570 dB/cm, 132,895.9509 dB/cm and 130,043.14660 dB/cm. It can be seen from the data that the peak loss is greatly affected by the hole radius. This is mainly due to the increment of the hole radius, which makes the gold film closer to the core and enhances the plasmon wave. When the radius is 0.52 *μm*, the loss is greater than other radius values, so the radius of Channel 1 is fixed at 0.52 *μm*.

**Figure 4:**
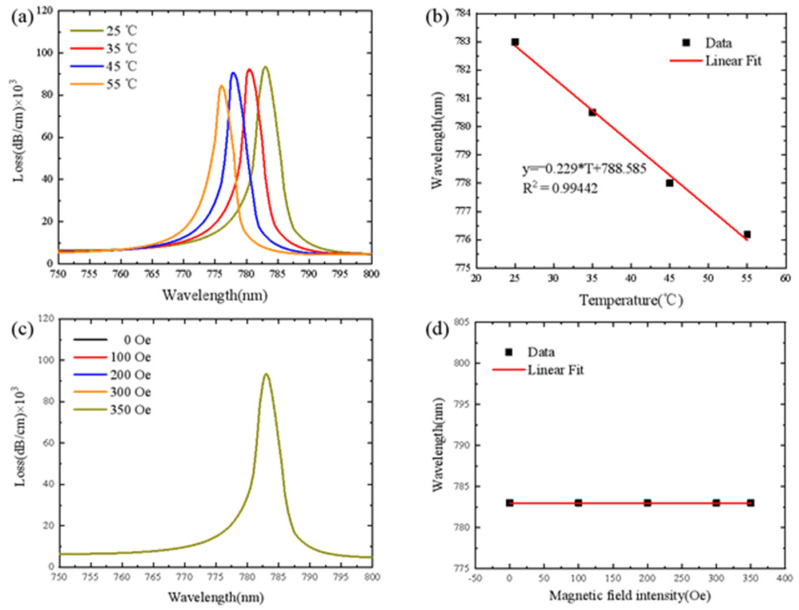
(**a**) Loss spectra of Channel 1 under different temperatures (magnetic field is fixed as 0 Oe). (**b**) Fitting results of resonance wavelength at different temperatures. (**c**) Loss spectra of Channel 1 under different magnetic field strength (temperature is fixed as 25 °C). (**d**) Fitting results of resonance wavelength at different magnetic field strength.

The loss spectrum of D-type optical fiber SPR sensor for Channel 1 at different temperatures is plotted in Figure 4a. The thickness of the deposited gold film is 50 nm and the radius of the filling hole is 0.52 *μm*. When the temperature is 25 °C, 35 °C, 45 °C and 55 °C, the resonance wavelengths are 783 nm, 780.5 nm, 778 nm and 776 nm, respectively. The peak loss values are 93,586.9451 dB/cm, 92,309.8663 dB/cm, 90,615.0833 dB/cm and 84,595.8848 dB/cm, respectively. With the increase in temperature, the resonance wavelength moves to shorter wavelengths, and the loss peak decreases. Figure 4b is the linear fitting curve of the resonance wavelength at different temperatures, which shows a good linear relationship. The temperature sensitivity of Channel 1 of D-type optical fiber SPR sensor is −0.229 nm/°C. As shown in Figure 4c, when the magnetic field intensity increases from 0 Oe to 350 Oe, the loss spectrum does not shift and the resonance wavelength position does not change. This is because PDMS is not affected by magnetic field intensity and the change of magnetic field intensity will not cause the shift of resonance wavelength of Channel 1. The fitting curve is shown in Figure 4d and the magnetic field sensitivity of Channel 1 is 0 nm/Oe.

**Figure 5:**
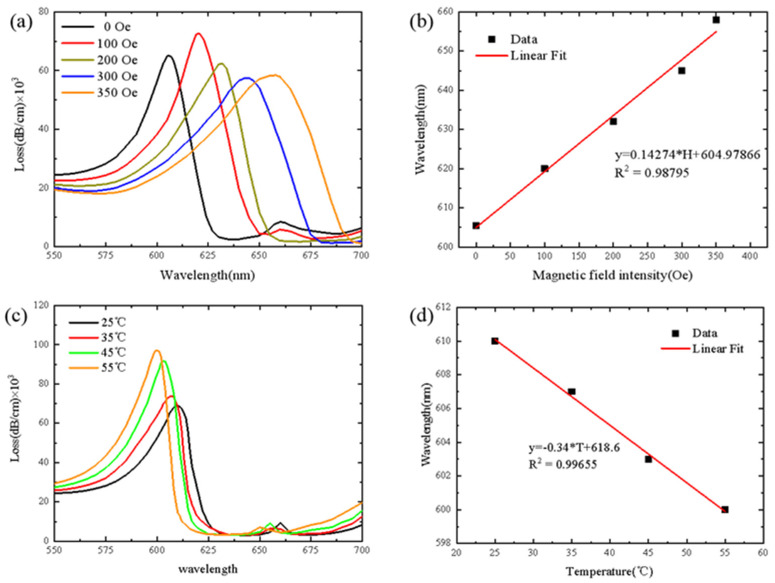
(**a**) Loss spectra of Channel 2 under different magnetic field strength (temperature is fixed as 25 °C). (**b**) Fitting results of resonance wavelength at different magnetic field strength. (**c**) Loss spectra of Channel 2 under different temperatures (magnetic field is fixed as 0 Oe). (**d**) Fitting results of resonance wavelength at different temperatures.

The loss spectrum of D-type optical fiber SPR sensor for Channel 2 under different magnetic field strength is shown in Figure 5a. When the magnetic field intensity is 0 Oe, the resonance wavelength is 605.5 nm and the loss peak is 64,903 dB/cm. When the magnetic field intensity is 350 Oe, the resonance wavelength is 658 nm and the loss peak is 58,572.5 dB/cm. As shown in Figure 5a, with the increase in magnetic field strength, the resonance wavelength moves to longer wavelengths and the loss peak value decreases. Figure 5b shows the fitting linear curve of resonance wavelength under different magnetic field strength. The magnetic field sensitivity of D-type SPR sensor for Channel 2 is 0.14274 nm/Oe. As shown in Figure 5c, when the temperature changes from 25 °C to 55 °C, the loss spectrum at the wavelength of 640 nm shifts to 670 nm. When the temperature is 25 °C, the resonance wavelength is 608.5 nm and the peak loss is 69,219.79362 dB/cm. When the temperature is 55 °C, the resonance wavelength is 601 nm and the peak loss is 97,289.93465 dB/cm. With the increase in temperature, the loss spectrum shifts to the left, the resonance wavelength moves to shorter wavelengths and the loss peak value decreases. The fitting curve is shown in Figure 5d and the temperature sensitivity of Channel 2 is −0.34 nm/°C.

According to the above analysis, the temperature sensitivity and magnetic field sensitivity of Channel 1 are −0.229 nm/°C and 0 nm/Oe, respectively. The temperature sensitivity and magnetic field sensitivity of Channel 2 are −0.34 nm/°C and 0.14274 nm/Oe, respectively. By substituting the four sensitive data into Equation (5), the following results are obtained:

(7) $$\left(\begin{array}{c} \Delta T\\ \Delta H \end{array}\right)={\left(\begin{array}{cc} -0.229 & 0\\ -0.34 & 0.14274 \end{array}\right)}^{-1}\left(\begin{array}{c} \Delta \lambda_{1}\\ \Delta \lambda_{2} \end{array}\right)=\left(\begin{array}{cc} -4.3668 & 0\\ -10.4045 & 7.0077 \end{array}\right)\left(\begin{array}{c} \Delta \lambda_{1}\\ \Delta \lambda_{2} \end{array}\right)$$

Obviously, when the temperature and the resonance wavelength offset of the magnetic field are known, the variation of the magnetic field and the temperature in the external environment can be obtained. This indicates that the proposed sensor can measure both magnetic field and temperature, therefore solving the cross-sensitivity problem.

To make a comprehensive comparison, Table 1 lists more specific details of the proposed sensor in this work with literatures.

## 4. Conclusions

To conclude, a magnetic field sensor based on temperature self-reference is designed and analyzed. The RI of magnetic fluid will be affected by both magnetic field and temperature. When measuring the magnetic field by filling with the magnetic fluid, the temperature variable will have an impact on the measurement results. The RI of PDMS only decreases with increasing temperatures, and it is not affected by the magnetic field intensity. Therefore, we can calculate the variation of temperature according to the filled PDMS, and then eliminate the influence of temperature and further infer the real magnetic field. In addition, by optimizing the radius of the filling hole and the thickness of the gold film, the D-type PCF sensor with magnetic field sensitivity of 0.14274 nm/Oe and temperature sensitivity of −0.229 nm/°C can be achieved. This simple yet effective device is conducive to the wide applications of the proposed sensors in power, medicine and many other fields.

## Figures and Tables

**Table 1:** Performance comparison of various optical fiber magnetic field sensors.

Sensor Structure	Magnetic Sensitivity (nm/Oe)	Detection Range (Oe)
PCF [17]	0.09246	0–100
S-taper fiber [18]	−0.03464	40–160
Mach-Zehnder [19]	0.04078	0–250
This work	0.14274	0–350
